# Interaction of β_L_- and γ-Crystallin with Phospholipid Membrane Using Atomic Force Microscopy

**DOI:** 10.3390/ijms242115720

**Published:** 2023-10-29

**Authors:** Nawal K. Khadka, Preston Hazen, Dieter Haemmerle, Laxman Mainali

**Affiliations:** 1 Department of Physics, Boise State University, Boise, ID 83725, USA; nawalkhadka@boisestate.edu (N.K.K.); dieterhaemmerle@u.boisestate.edu (D.H.); 2 Biomolecular Sciences Graduate Program, Boise State University, Boise, ID 83725, USA; prestonhazen@u.boisestate.edu

**Keywords:** phospholipid membrane, cholesterol, β_L_- and γ-crystallin, membrane-crystallin interactions, AFM, topographical images, transmembrane defects, semi-transmembrane defects, cataract

## Abstract

Highly concentrated lens proteins, mostly β- and γ-crystallin, are responsible for maintaining the structure and refractivity of the eye lens. However, with aging and cataract formation, β- and γ-crystallin are associated with the lens membrane or other lens proteins forming high-molecular-weight proteins, which further associate with the lens membrane, leading to light scattering and cataract development. The mechanism by which β- and γ-crystallin are associated with the lens membrane is unknown. This work aims to study the interaction of β- and γ-crystallin with the phospholipid membrane with and without cholesterol (Chol) with the overall goal of understanding the role of phospholipid and Chol in β- and γ-crystallin association with the membrane. Small unilamellar vesicles made of Chol/1-palmitoyl-2-oleoyl-sn-glycero-3-phosphocholine (Chol/POPC) membranes with varying Chol content were prepared using the rapid solvent exchange method followed by probe tip sonication and then dispensed on freshly cleaved mica disk to prepare a supported lipid membrane. The β_L_- and γ-crystallin from the cortex of the bovine lens was used to investigate the time-dependent association of β_L_- and γ-crystallin with the membrane by obtaining the topographical images using atomic force microscopy. Our study showed that β_L_-crystallin formed semi-transmembrane defects, whereas γ-crystallin formed transmembrane defects on the phospholipid membrane. The size of semi-transmembrane defects increases significantly with incubation time when β_L_-crystallin interacts with the membrane. In contrast, no significant increase in transmembrane defect size was observed in the case of γ-crystallin. Our result shows that Chol inhibits the formation of membrane defects when β_L_- and γ-crystallin interact with the Chol/POPC membrane, where the degree of inhibition depends upon the amount of Chol content in the membrane. At a Chol/POPC mixing ratio of 0.3, membrane defects were observed when both β_L_- and γ-crystallin interacted with the membrane. However, at a Chol/POPC mixing ratio of 1, no association of γ-crystallin with the membrane was observed, which resulted in a defect-free membrane, and the severity of the membrane defect was decreased when β_L_-crystallin interacted with the membrane. The semi-transmembrane or transmembrane defects formed by the interaction of β_L_- and γ-crystallin on phospholipid membrane might be responsible for light scattering and cataract formation. However, Chol suppressed the formation of such defects in the membrane, likely maintaining lens membrane homeostasis and protecting against cataract formation.

## 1. Introduction

Lens cytoplasmic proteins, mostly crystallin proteins, are responsible for maintaining the structure, transparency, and refractivity of the vertebrates’ eye lens for the length of their lives. Crystallin proteins constitute almost 90% of total soluble lens proteins and maintain uniform concentration gradients to preserve lens properties. The crystallin proteins in eye lenses are highly concentrated and packed compactly in short-range spatial order to enhance lens transparency and maintain an appropriate refractive index [1,2]. Eye lens crystallins are divided into two major superfamilies, α-crystallin and βγ-crystallin where βγ-crystallin constitutes around 50% of the cytoplasmic lens proteins [3]. α-crystallins, comprised of αA and αB subunits, are small heat shock proteins that may prevent the aggregation of other partially folded proteins and opacity of the lens by acting as a molecular chaperone [4]. One of the characteristic structural features of the βγ-crystallin superfamily is intercalated double β-sheet Greek key motifs in their two domains connected by 8–10 amino acid inter-domain connecting peptide [5]. The seven subunits of β-crystallin, whose molecular weights range from approximately 23–28 kDa, are obtained as a homogeneous mixture of dimer or higher oligomeric forms in their native states [6] with molecular weights from 50 kDa–200 kDa [7]. In contrast, γ-crystallin exists as monomers with molecular weights of approximately 21 kDa [8,9]. Due to the lack of turnover, these structural proteins demand extreme stability to maintain the functional lens; however, with aging, the stability of these crystallins deteriorates, disrupting the orderly arrangement of protein packing.

Several hypotheses have been proposed for cataractogenesis; however, the mechanism for cataract formation is unclear. Transparency is the principal property of the lens and is reduced by the scattering of light from lens components. Significant studies have been performed in this area, and sources of light scattering include multilamellar bodies (MLBs) containing the core of crystallins proteins covered by multiple lipid bilayers [10,11,12,13], aggregation of crystallins [14,15], and crystallin-membrane association [16,17,18]. It has been reported from protein density studies that the cytoplasm of transparent lens nuclei has uniform protein packing [10]. In contrast, dense protein packing was observed in cataractous lens cytoplasm [10]. It has been reported that MLBs are large particles suggested to be significant sources of light scattering in human lens nuclei, where the protein packing in MLB cores during aging becomes increasingly different from the adjacent cytoplasm [10,11,12] supporting the hypothesis that with aging and cataract development, the light scattering is increased by MLBs [10].

It has been reported that with age and cataract formation, β- and γ-crystallin bind with the lens membrane [19,20,21,22] or with other lens proteins, forming high-molecular-weight (HMW) proteins [23,24,25,26], which further associate with the fiber cell plasma membrane [16,20,27,28], accompanied by light scattering and cataract formation [16,17,18]. The content of β- and γ-crystallin fragments in HMW proteins is more significant in the cataractous lenses compared to age-matched normal lenses [23]. It is still unclear how HMW protein originates and binds with the membrane together with light scattering and cataract formation [23,25,29,30,31,32]. With the increase in age, the level of the soluble lens proteins declines with the corresponding increase in water-insoluble proteins [33]. Primarily, all water-insoluble crystallins in the aged human lens bind to the membranes [17], which is highly believed to contribute to the onset of nuclear cataracts by occluding membrane pores and creating a barrier to diffusion [20,28,34]. The strongly bounded protein content on the membrane, obtained via multiple washes with denaturing agents, contains a majority of β- and γ-crystallin and is found to increasingly correlate with aging, indicating a tight binding of β- and γ-crystallin with the lens membrane [21]. The tight binding of the β- and γ-crystallin to the lens membrane will likely alter membrane structure, forming aggregates responsible for light scattering and cataract formation.

Multiple studies on the interaction of α-crystallin with model and plasma membranes have been performed by our lab [32,35,36,37,38,39,40] and several others [41,42,43,44,45]. However, knowledge of the interactions of β- and γ-crystallin with membranes is limited. Moreover, the study of the role of cholesterol (Chol) in such interactions is almost non-existent. Recently, we used the electron paramagnetic resonance (EPR) spin-labeling method and showed that lipid and Chol composition strongly modulates the binding of β_L_-crystallin with the models of animal and human eye lens–lipid membranes [46]. Previously, Zhu et al. [22] used a fluorescence approach and performed the vesicle binding experiment with the vesicles prepared from di-hydro sphingomyelin and found that β-crystallin affected the headgroup order. It has been reported that the physical properties of the fiber cell membranes in the lens nucleus change significantly with age, and β-crystallin may be directly responsible for modulating membrane properties in the lens [22]. The association of βB1 crystallin to the bovine lens plasma membrane was found when analyzed by sodium dodecyl sulfate-polyacrylamide gel electrophoresis (SDS-PAGE) [19]. Specific interaction of γE and γF-crystallins with the intrinsic membrane protein MIP/Aquaporin-0 was evidenced earlier [47,48]. Previous studies show that the content of β- and γ-crystallin in membranes isolated from the outer cortex, barrier, inner, and core regions of human lenses become increasingly membrane-bound with age [21]. Likely, such binding will significantly compromise access to membrane pores, creating a barrier that blocks the diffusion of antioxidants and initiates cataract formation [49,50].

The β- and γ-crystallins in the eye lens are enclosed with the membrane composed of sphingolipids (sphingomyelin (SM) and di-hydro SM), phospholipids (PLs), Chol, and intrinsic membrane proteins [32,51]. The major headgroups of PLs found in lens membrane are phosphatidylcholine (PC), phosphatidylserine (PS), and phosphatidylethanolamine (PE). With increasing age, PC in the human lens membrane decreases with the corresponding increase in sphingolipids [52,53]. Additionally, Chol content increases significantly with age and is greater in nuclear membranes than in cortical membranes [54,55]. The composition of the lens lipids varies between animal species; shorter-life-span mammals have PC as the dominant lipid, while sphingolipids are dominant in longer-life-span mammals [56]. The acyl chains in the lipids in the lens membrane are composed primarily of palmitic (16:0, P) and oleic (18:1-cis, O) [56,57,58]. The lipid (PLs and sphingolipids) composition of animal and human lens membranes has been previously characterized; bovine lens membranes consist of 31% PC, 27% SM, 2% dihydro-SM, 15% PS, and 22% PE, whereas the 60-year-old human lens membrane consists of 11% PC, 19% SM, 47% dihydro-SM, 8% PS, and 15% PE [56]. The Chol/lipid molar ratios for two-year-old bovine cortical and nuclear membranes are reported to be 0.7 and 1.9, respectively [59]. For the transparent human lens membrane of the lower age group (0–20 years), the Chol/lipid molar ratios for cortical and nuclear membranes are 0.63 and 0.71, respectively, whereas for the higher age group (61–70 years), the Chol/lipid molar ratios for cortical and nuclear membranes are 1.8 and 4.4, respectively [55,60]. In the cataractous human lens membrane of the higher age group (61–70 years), the Chol/lipid ratios are significantly decreased to 1.14 and 1.45 for cortical and nuclear membranes, respectively, compared to age-matched transparent human lens membrane [60]. To investigate the interaction of β- and γ-crystallin with the membrane, in the study proposed here, we prepared the model membrane made of 1-palmitoyl-2-oleoyl-sn-glycero-3-phosphocholine (POPC) with varying Chol content to mimic the lens membrane’s lipid composition.

We recently developed the atomic force microscopy (AFM) approach to investigate the high-Chol-containing membranes relevant to eye lens membrane [61,62]. We also developed the AFM approach to investigate the interaction of α-crystallin with the phospholipid membrane and found a strong time-dependent association of α-crystallin to the membrane [35]. Most of the studies on cataract formation are connected with the posttranslational modification (e.g., deamidation. oxidation, and truncation) of crystallins [15,63]; however, the Chol composition in lens membrane changes significantly with age and cataract formation [55,60,64] and how change in Chol composition in membrane influence interaction of β-and γ-crystallin with membrane is unclear. In the present work, we investigated the time course interaction of β_L_-and γ-crystallin with the phospholipid membrane with varying Chol content and modulation of membranes’ mechanical properties by β_L_-crystallin using AFM where we have used native β_L_-crystallin, which consists different proportion of β-crystallin subunits (βB1, βB2, βB3, βA1, βA2, βA3, βA4) [8] resembling in vivo β_L_-crystallin composition and native γ-crystallin which consist different proportion of γ-crystallins (γA-, γB-, γC-, γD-, γE-, γF-, γN-, and γS) [65,66] resembling in vivo γ-crystallin composition. To our knowledge, the study reported here demonstrates the AFM approach for the first time to study the interaction of β_L_- and γ-crystallin with the membrane in the absence and presence of Chol. Our study shows that β_L_- and γ-crystallin strongly interact with the membrane-forming semi-transmembrane and transmembrane defect, and such defects might be responsible for light scattering and cataract formation. However, Chol inhibits the formation of such defects, likely maintaining lens membrane homeostasis and protecting against cataract formation.

## 2. Results

### 2.1. Interaction of β_L_-Crystallin with Membrane

**Figure 1:**
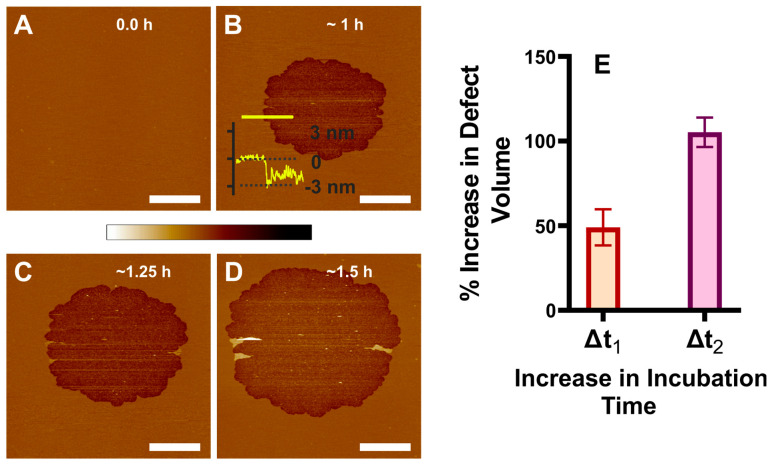
Interaction of β_L_-crystallin with POPC membrane. (**A**) POPC-only membrane. (**B**–**D**) POPC membrane interaction with β_L_-crystallin at different time intervals, as indicated in the figure. Almost all circular membrane defects are formed resulting from the interaction of β_L_-crystallin with the membrane. β_L_-crystallin forms a semi-transmembrane defect when it interacts with the membrane. The area of the semi-transmembrane defect increases with the incubation time. The height profile of the yellow line drawn across the membrane defect interface is shown in (**B**). (**E**) The percentage increase in semi-transmembrane defect volume with increased incubation time when β_L_-crystallin was incubated with Chol/POPC membrane at a mixing ratio of 0. The Δt_1_ represents an increase in incubation time from 1 h to 1.25 h, and Δt_2_ represents an increase in incubation time from 1 h to 1.5 h. Image scale: 5 µm; z-scale: −10 nm to 8 nm.

By capturing the topographical images, we investigated the time course association of the β_L_-crystallin with POPC supported lipid membrane (SLM). After confirming the formation of a defect-free membrane, as shown in Figure 1A, 0.025 mg/mL β_L_-crystallin solution in buffer B was dispensed on the SLM via an inlet in the AFM fluid cell using a syringe pump. The time course association of β_L_-crystallin to the membrane is shown in Figure 1B–D. The incubation time was set to 0 immediately after the 0.025 mg/mL β_L_-crystallin was dispensed to the defect-free membrane. β_L_-crystallin oligomers did not uniformly distribute on the membrane; instead, they clustered together as an aggregate, forming the defect, as shown in Figure 1B–D. Interestingly, the edge of the circular-shaped defect has a depth of 2.03 ± 0.36 nm (see Figure 1B for representative height profile). Considering that the thickness of the hydrated membrane for a POPC membrane is 3.9 ± 0.33 nm [62,67] excluding the water layer, the defect’s depth spans only a single layer of mica-supported POPC membrane, suggesting the formation of semi-transmembrane defect. No additional membrane defects were detected within the field of view with increased incubation time; however, the area of the semi-transmembrane defect increases, as shown in Figure 1B,D. Although no other visible patches are detected within the imaged region and incubated time, small bright objects were observed within the semi-transmembrane defect area. The area covered by the semi-transmembrane defects was quantified by image analysis. Based on the area occupied by the semi-transmembrane defect resulting from the association of β_L_-crystallin with the membrane, the size of these defects increases to 49 ± 10% and 105 ± 9% with an increase in incubation from ~1 h to ~1.25 h and from ~1 h to ~1.5 h, respectively. Figure 1E shows the percentage increase in semi-transmembrane defect volume plotted with an increase in incubation time when β_L_-crystallin was incubated with POPC membrane. The semi-transmembrane defect volume was estimated based on the area of the defect and the depth of the defect. Δt_1_ represents the incubation time interval from 1 h to 1.25 h, and Δt_2_ represents the incubation time interval from 1 h to 1.5 h. The Mann–Whitney test shows that the percentage increase in semi-transmembrane defect volume between Δt_1_ and Δt_2_ is significantly different (*p* < 0.1). Such semi-transmembrane defects might contribute to light scattering and cataract formation, and an increase in the size of the transmembrane defects suggests increased light scattering. Previously, we have investigated the association of α-crystallin with POPC SLM and found that α-crystallin oligomers did not uniformly associate with the membranes; instead clustered as an aggregate in the membrane forming α-crystallin–membrane complexes with increasing size as incubation time increased [35]. The study reported here (Figure 1) shows that β_L_-crystallin interacts with the membrane forming the semi-transmembrane defects, and with increasing incubation time, the size of such defects increases, suggesting that β_L_- and α-crystallin modulate with the membrane differently.

### 2.2. Effect of Chol in Membrane-β_L_-Crystallin Association

**Figure 2:**
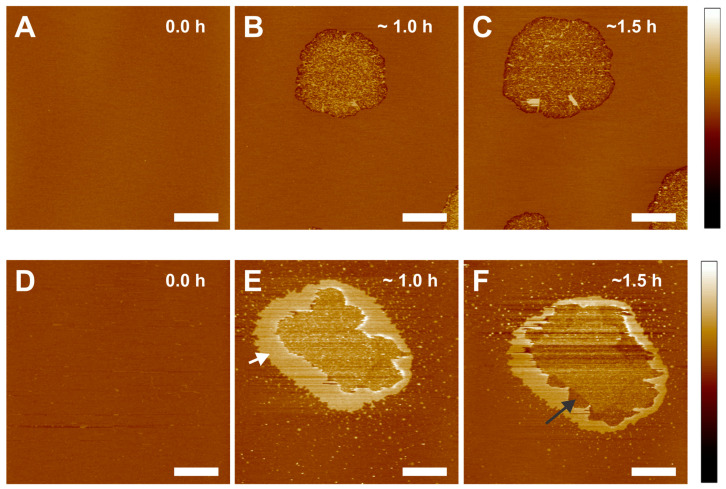
Effect of Chol on the interaction of β_L_-crystallin with Chol/POPC membrane at mixing ratios of 0.3 and 1. The top panels (**A**–**C**) are the images of the time course interaction of β_L_-crystallin with Chol/POPC membrane at a mixing ratio of 0.3. The bottom panels (**D**–**F**) are the images of the time course interaction of β_L_-crystallin with Chol/POPC membrane at a mixing ratio of 1. (**A**) Chol/POPC membrane without β_L_-crystallin. (**B**,**C**) Images of Chol/POPC membrane with the addition of 0.025 mg/mL of β_L_-crystallin and incubating for ~1 h and ~1.5 h, respectively. The size of β_L_-crystallin induced defect increases with time. The darker color at the edge of the defects shows a trench at the boundary of the β_L_-crystallin induced defect. The core consists of granular protrusion immediately after the trench, surging above the membrane surface. (**D**) Chol/POPC membrane without β_L_-crystallin. (**E**,**F**) Images of Chol/POPC membrane after adding 0.025 mg/mL of β_L_-crystallin solution and incubating for ~1 h and ~1.5 h, respectively. The β_L_-crystallin interaction with the membrane formed several different structures. Around the boundary, the raised structures with flat–smooth top surfaces are formed. In the center, the topography is granular, with an intermediate height between the membrane and the boundary region. These structures deteriorate with an increment in time, as seen from the darker shades in (**F**). Image scale: 10 μm; z-scale: top panel from −10 nm to 8 nm and bottom panel from −16 nm to 14 nm.

Eye lens membranes consist of extremely high levels of Chol content, and the Chol content increases with age and is greater in nuclear membranes compared to cortical membranes [54,55]. The role of Chol in the association of β_L_-crystallin with the lens membrane is unclear. We prepared the SLM containing Chol with a Chol/POPC mixing ratio of 0.3 and 1 and investigated the association of β_L_-crystallin with the Chol-containing membrane. The topographic images of the Chol/POPC membrane at a mixing ratio of 0.3 are displayed in the top row—Figure 2A–C, where Figure 2A is the Chol/POPC membrane—before adding 0.025 mg/mL of β_L_-crystallin, and Figure 2B,C are the time course association of β_L_-crystallin with the membrane. Similar to the POPC-only membrane (Figure 1), circular patches were obtained in Chol/POPC membrane at a mixing ratio of 0.3 when incubated with β_L_-crystallin; however, the patch consisting of granular structures protruding above the membrane. On the other hand, at the boundary of the patches, we observed a trench of almost 2.08 ± 0.42 nm depth, similar to the POPC membrane. The patch size increased, but no additional patches were formed with increased incubation time. These results show that at a Chol/POPC mixing ratio of 0.3, the semi-transmembrane defect is observed only on the boundary of patches. The height of the patches increases inside the boundary (i.e., patches are shifted toward or above the membrane surface inside the boundary), suggesting that Chol suppresses the formation of semi-transmembrane defects when β_L_-crystallin is incubated with the Chol-containing membrane. In the absence of Chol, semi-transmembrane defects were observed in all the patch regions of the membrane.

Similarly, the topographic images of the Chol/POPC membrane at a mixing ratio of 1 without the incubation of β_L_-crystallin are shown in Figure 2D, and with the incubation of β_L_-crystallin is shown in Figure 2E,F. The smooth Chol/POPC membrane at a mixing ratio of 1 in Figure 2D evolves distinctly after incubation with β_L_-crystallin, as shown in Figure 2E, compared to the Chol/POPC membranes at mixing ratios of 0 and 0.3 (Figure 1B–D and Figure 2B,C). After ~1 h of β_L_-crystallin incubation, the smooth membrane developed a large non-circular patch with elevated height, as indicated by the white arrow in Figure 2E, surrounded by tiny bright patches. The large patch comprised two distinct features: a raised boundary of an almost continuous smooth patch with 4.49 ± 0.49 nm height above the membrane and a granular core with a height slightly lower than that of a smooth boundary. The granular core was similar to the patches obtained in the Chol/POPC membrane at a mixing ratio of 0.3 with β_L_-crystallin incubation. The topographic image obtained at around 1.5 h of β_L_-crystallin incubation (Figure 2F) shows the smooth patches formed earlier eroded, as indicated by a black arrow in Figure 2F, and formed random trenches. However, the trench depth was lower than the granular patch’s height. Also, the number of tiny patches formed around the large patches decreased as the incubation time increased. These results show that the increasing Chol content in the membrane, although it exhibits defects, suppresses the formation of semi-transmembrane defects in the membrane when incubated with β_L_-crystallin. This suppression of semi-transmembrane defect formation suggests that Chol protects the integrity of the membrane by suppressing the association of β_L_-crystallin with the membrane and might protect against cataract formation. Our recent study [46] using the EPR spin-labeling method showed that Chol reduces the binding affinity of β_L_-crystallin to the models of animal and human eye lens-lipid membranes. Previously, our results show that Chol inhibits α-crystallin binding to Chol/POPC membrane, and a higher level of inhibition was observed with increasing Chol content [36].

### 2.3. γ-Crystallin Association with Membrane

**Figure 3:**
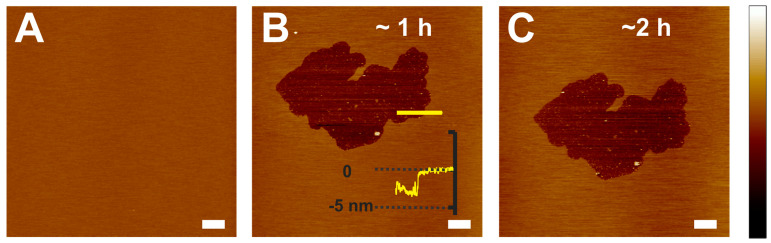
Interaction of γ-crystallin with POPC membrane. (**A**) POPC-only membrane. (**B**,**C**) POPC membrane interaction with γ -crystallin in a different time interval, as indicated in the figure. Unlike the effect of β-crystallin, γ-crystallin forms irregular-shaped defects while interacting with the POPC membrane. Additionally, the depth of the defects formed by the effect of γ-crystallin, as shown in (**B**), is close to transmembrane thickness. The area of the transmembrane defect slightly increases with incubation time. Image scale: 5 µm; z-scale: −12 nm to 10 nm.

The time course association of γ-crystallin with the POPC membrane was monitored by capturing topographical images using AFM. Figure 3A shows the control POPC membrane before adding γ-crystallin, and Figure 3B,C shows the association of γ-crystallin with the membrane at different incubation times, as indicated. γ-crystallin associated with the POPC membrane forms an irregular defect at the interaction site. The depth of the defect, i.e., γ-crystallin-membrane-associated patch, is 3.5 ± 0.29 nm, which corresponds closely to the membrane thickness of the POPC membrane (see Figure 3B for a representative height profile). This defect depth suggests the formation of transmembrane defects by γ-crystallin, unlike semi-transmembrane defects developed by β_L_-crystallin association with POPC membrane (Figure 1). Such transmembrane defects might contribute to light scattering and cataract formation. Based on the area occupied by the transmembrane defects resulting from the association of γ-crystallin with the membrane, the size of transmembrane defects increases by 2.8 ± 1.1% with an increase in incubation from ~1 h to ~2 h. These results suggest that no significant increase in the size of the transmembrane defect was observed with an increase in incubation time, whereas in the case of β_L_-crystallin association with POPC membrane, the size of semi-transmembrane defects increases significantly with an increase in incubation time, as discussed in Section 2.1.

### 2.4. Chol Supressess Membrane-γ-Crystallin Association and Membrane Defect Formation

**Figure 4:**
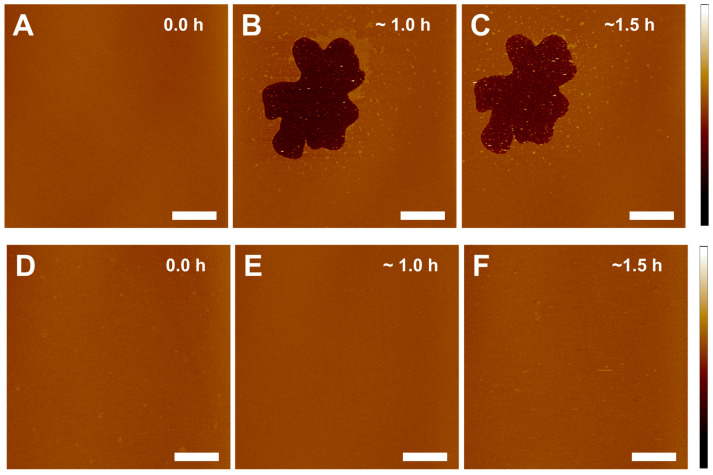
Effect of Chol on the interaction of γ-crystallin with Chol/POPC membrane at mixing ratios of 0.3 and 1. The top panels (**A**–**C**) are the images of the time course interaction of γ-crystallin with Chol/POPC membrane at a mixing ratio of 0.3. The bottom panels (**D**–**F**) are the images of the time course interaction of γ-crystallin with Chol/POPC membrane at a mixing ratio of 1. (**A**) Chol/POPC membrane without γ-crystallin. (**B**,**C**) Images of Chol/POPC membrane with the addition of 0.025 mg/mL of γ-crystallin and incubating for ~1 h and ~1.5 h, respectively. The γ-crystallin induced irregular defects on the membrane, and the defect remains almost the same size with increases in time. (**D**) Chol/POPC membrane without γ-crystallin. (**E**,**F**) Images of Chol/POPC membrane after adding 0.025 mg/mL of γ-crystallin solution and incubating for ~1 h and ~1.5 h, respectively. Interestingly, at this high Chol content (Chol/POPC mixing ratio of 1), 0.025 mg/mL γ-crystallin solution did not form any defects on the membrane. Image scale: 10 μm; z-scale: −12 nm to 10 nm.

Increasing Chol content with age in the eye lens membrane makes it very intriguing to study the interaction of γ-crystallin with Chol-containing membranes. We studied the outcome of Chol in the association of γ-crystallin with the lens membrane. The effect of Chol in an association of γ-crystallin with POPC membrane is displayed in Figure 4. The top row image shows the γ-crystallin association with Chol/POPC membrane at a mixing ratio of 0.3, and the bottom row shows the incubation of γ-crystallin with Chol/POPC membrane at a mixing ratio of 1. The smooth surface of the Chol/POPC membrane at a mixing ratio of 0.3 without the addition of γ-crystallin is displayed in Figure 4A. After a 1 h incubation of γ-crystallin with Chol/POPC membrane at a mixing ratio of 0.3, an irregularly shaped defect was observed, as shown in Figure 4B. The depth of such a patch was 4.38 ± 0.78 nm, which is close to the membrane thickness. On further incubation, the shape/size of the patch remained almost identical, as shown in Figure 4C, similar to the effect seen when γ-crystallin was incubated with Chol free membrane (Section 2.3). We also estimated the change in transmembrane defect volume when γ-crystallin was incubated with Chol/POPC membrane at mixing ratios of 0 and 0.3; however, we did not find a significant change in transmembrane defect volume when the incubation time increased from 1 h to ~2 h (for Chol/POPC mixing ratio of 0) and from 1 h to 1.5 h (for Chol/POPC mixing ratio of 0.3). However, when γ-crystallin was incubated with a membrane having increased Chol content, i.e., at Chol/POPC mixing ratio of 1, γ-crystallin incubation did not display any association site within our region and imaging time (Figure 4D,E). These results show that higher Chol suppresses the association of γ-crystallin to the membrane, preventing the formation of transmembrane defects and which might protect against light scattering and cataract formation.

### 2.5. Mechanical Properties of the Membrane–Crystallin System

**Figure 5:**
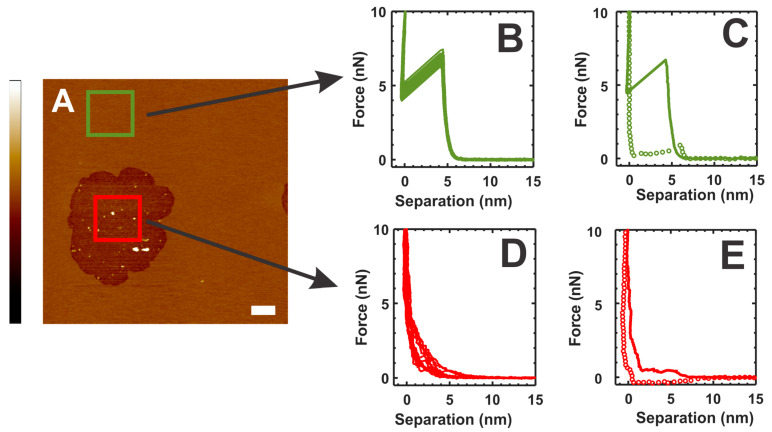
(**A**) Interaction of β_L_-crystallin with POPC membrane at around 1.25 h incubation time where green square represents a defect-free membrane region where the force curves are collected, and red square represents the semi-transmembrane defect regions where the force curves are collected. (**B**) Collection of approach force curves obtained in the defect-free membrane regions (no β_L_-crystallin–membrane interaction) identified by the green square in (**A**). (**C**) Representative complete force curve of the defect-free membrane region (identified by the green square in (**A**) with approach curve in solid green line and retract curve in green circles, respectively. These force curves show typical bilayer penetration by the AFM tip, and breakthrough events are obtained. (**D**) Collection of approach force curves obtained in the semi-transmembrane defect regions (identified by the red square in (**A**)) resulting from the interaction of β_L_-crystallin with the membranes. (**E**) Representative complete force curve of the semi-transmembrane defect region (identified by the red square in (**A**)) with approach and retract curves shown in solid red line and red circles, respectively. The force curves in D represent a loss of membrane elastic property as indicated by no breakthrough events. Image scale: 10 µm; z-scale: −12 nm to 10 nm.

To investigate the mechanical properties of the membrane with β_L_-crystallin association, we collected the force curves in defect-free membrane region (no β_L_-crystallin membrane association) shown by the green square in Figure 5A and in the semi-transmembrane defect region resulting from the interaction of β_L_-crystallin with the membrane shown by the red square in Figure 5A. The representative force curves collected in the defect-free membrane region (identified by the green square in A) are displayed in Figure 5B, showing the typical force curves of the membrane with breakthrough events. The average breakthrough force in defect-free membrane regions estimated from three independent experiments is 5.11 ± 1.35 nN. A similar breakthrough force was obtained earlier in α-crystallin-free membrane regions [35]. The elastic nature of the membrane is maintained as indicated by the clear breakthrough events in the representative force curves shown in Figure 5B. The representative complete force curves collected in the defect-free membrane region are shown in Figure 5C, where solid green line represents the approach force curve and dotted green circles represent the retract force curve. The representative force curves collected in semi-transmembrane defect regions (identified by the red square in A) are displayed in Figure 5D, showing the loss of the typical elastic nature of the membrane. The representative complete force curves collected in the transmembrane defect region are shown in Figure 5E, where a solid red line represents the approach force curve and dotted red circles represent the retract force curve. As shown in Figure 5D, the force curves obtained in the semi-transmembrane defect region (β_L_-crystallin membrane association region) did not display typical membrane force curves with breakthrough events within the set force threshold limit, suggesting the loss of membrane elasticity. Previously, we have observed similar force curves without breakthrough events within the defined peak force threshold and loss of typical membrane force curve behavior in the α-crystallin-submerged membrane region and the close vicinity of the α-crystallin-associated membrane regions, indicating the loss of membrane elasticity [35].

Furthermore, the modified Hertz model can be used to describe the force curves in the membrane obtained using AFM [35,68]:

(1) $$F=\frac{16}{9}ER^{1/2}\delta^{3/2}\left(1+0.884\rho +0.781\rho^{2}+0.386\rho^{3}+0.0048\rho^{4}\right)$$

where *E* is Young’s Modulus and is a measure of membrane elasticity, *δ* is indentation depth defined by *D-s*, with *D* being the distance between mica to the initial point of contact by AFM tip to the membrane, and *s* is the tip mica separation distance. Here ρ is a dimensionless parameter defined by $\sqrt{R\delta }/h$, where *R* is the tip-end radius of the AFM tip and *h = D-t_w_*, with *t_w_* being the water layer thickness assumed to be 2 nm [69]. As described in our previous studies [35], we have fitted the elastic regime (i.e., approach force curves until 80% of breakthrough force) of the force curves (representative force curves are shown in Figure 5B) using Equation (1) to estimate *E*. The estimated value of *E* in the defect-free membrane region obtained from the average of three independent experiments is 26.4 ± 9.0 MPa. Similar *E* values were obtained earlier in the α-crystallin-free membrane region [35]. We could not estimate the *E* value from the obtained force curves in semi-transmembrane defect regions because of the loss of typical elastic behavior of the membrane. Previously, we could not estimate the *E* value from the non-elastic nature of force curves obtained in the α-crystallin-submerged membrane region and in the vicinity of the α-crystallin-membrane-association region [35].

Based on the study reported above about the loss of elastic behavior of the force curves resulting in the loss of membrane elasticity in the β_L_-crystallin membrane association region (i.e., semi-transmembrane defect regions) and our earlier studies [35] on loss of membrane elasticity in α-crystallin membrane association regions, we expect similar force curves with loss of elastic behavior in the β_L_-crystallin membrane association region at Chol/POPC mixing ratios of 0.3 and 1 and the γ-crystallin membrane association region at Chol/POPC mixing ratios of 0 and 0.3; however, further research is needed in this area.

### 2.6. Distribution of β_L_- and γ-Crystallin in the Absence of Membrane

**Figure 6:**
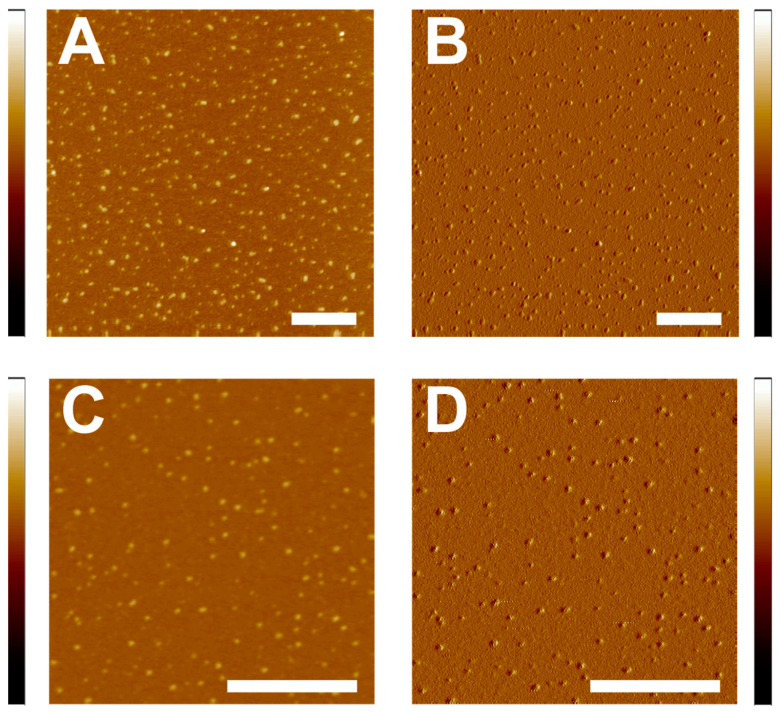
Distribution of βL- and γ-crystallin on clean mica disk scanned in air. (**A**,**B**) Topographical and peak force error images of βL-crystallin, respectively. (**C**,**D**) Topographical and peak force error images of γ-crystallin, respectively. Image scale: 0.5 µm; z-scale: left from −8 nm to 6 nm and right from −0.20 nN to 0.15 nN.

Figure 6 displays the representative distribution of electrostatically associated β_L_- and γ-crystallin on the mica surface. Figure 6A,B are the topographical and peak force error images of the β_L_-crystallin, respectively, while Figure 6C,D are the topographical and peak force error images for γ-crystallin, respectively. The β_L_-crystallin in the bovine lens is dimer and trimer [8], and γ-crystallin exist as monomers. Several factors, including tip size, setpoint force, pixel size, ambient condition, flattening of the molecules, etc., determine the actual size of the monomers/oligomers by AFM. However, our purpose is to determine the interaction of β_L_- and γ-crystallin with the membrane, and we did not measure the size of the monomers/oligomers using AFM. Relying on visual observation of the images, β_L_-crystallin displayed a slightly poly-dispersed distribution on the mica surface (Figure 6A,B). Comparison of this distribution to the granular structures protruding above the membrane immediately after the trench (Figure 2B,C) suggests that in the granular structure, β_L_-crystallins might be submerged in the membrane, which are localized in the membrane regions, whereas the slightly poly-dispersed distribution of β_L_-crystallin was observed throughout the mica surface (Figure 6A,B). Visual observations of the images (Figure 6C,D) display uniform distribution of γ-crystallins. Similar structures were demonstrated for native human γD-crystallin visualized using AFM [70].

We have determined the hydrodynamic radius (R_h_) and percentage of polydispersity (% Pd) by dynamic light scattering (DLS) for β_L_- and γ-crystallin. The R_h_ and % Pd estimated for β_L_-crystallin are 3.5 ± 0.2 nm and 11.6 ± 2.2, respectively, whereas the R_h_ and % Pd estimated for γ -crystallin are 2.3 ± 0.1 nm and 7.1 ± 1.6, respectively. A smaller % Pd value in γ -crystallin indicates that the size distribution is monodisperse, whereas a slightly larger % Pd value in β_L_-crystallin indicates that the size distribution is slightly polydisperse; similar size distribution is also visualized in the AFM images of β_L_- and γ-crystallin in the mica surface (Figure 6A–D). The higher R_h_ value of β_L_-crystallin compared to γ -crystallin indicates a larger size of β_L_-crystallin compared to γ-crystallin. Earlier studies show that β_L_-crystallin exists as a trimer (β_L1_) and dimer (β_L2_) [8] with an average molecular weight of the β_L_-crystallin subunit of 24.31 kDa, whereas the γ-crystallin exist as a monomer [65] with an average molecular weight of γ-crystallin of 21.04 kDa (see Section 4.2). Previous studies reported R_h_ values for γ-crystallin ranges of 2.3 nm to 2.5 nm [65] and R_h_ values for β_L2_-crystallin of 2.9 nm [71].

## 3. Discussion

All seven types of β-crystallin subunits (basic: βB1, βB2, βB3; acidic βA1, βA2, βA3, βA4) [6] and mostly γC-, γD-, and γS-crystallins [72] are obtained in the human lenses, where postnatally expressed γS synthesis increases with aging. While in the bovine lens, all seven β-crystallins [8] and γB-, γC-, γD-, γE-, γF-, and γS-crystallins are reported [65,66], for our experimental purpose, we did not identify the type of β- or γ-crystallin. We used low-molecular-weight β_L_-crystallin, primarily consisting of dimers and trimers, for all our experiments [8]. Despite having a close sequence homology, the β-crystallin involves intermolecular packing of domains forming oligomers, whereas γ-crystallin involves intramolecular packing with the N-terminal and C-terminal domains coming together to generate a compact monomeric globule [2].

In the Chol-free membrane, β_L_-crystallin induced sporadic circular defects with a depth of around half the bilayer thickness on the supported phospholipid membrane. This experiment is the first to exhibit the effect of β_L_-crystallin spanning only on the distal leaflet but not throughout the whole membrane, as shown by semi-transmembrane defects. Generation of such semi-transmembrane defects warrants β_L_-crystallin for the removal of lipids only from the distal leaflet of an SLM, which endorses the proximal leaflet’s acyl chain within the semi-transmembrane defects exposed to the water layer, which is energetically unfavored. Several mechanisms might have evolved to antagonize this energetically unfavored state. The excess β_L_-crystallin that did not participate in the interaction may have blanketed the exposed hydrophobic regions. The interaction of β_L_-crystallin with the exposed acyl chain also cannot be neglected, as the AFM imaging in later times introduces smaller bright objects within the defect area. Another possibility might be that the removed lipids interdigitate with the exposed hydrophobic regions. Such semi-transmembrane domains in several lipid types have been observed during the interaction of presynaptic protein α-synuclein [73,74].

The effect of the association of γ-crystallin with the POPC membranes results in the formation of transmembrane defects/pores. For pore formation, it has been suggested that the minimal number of pore-forming peptide units required is 3 to 10 [75]. Melittin, a pore-forming peptide, induced gradual membrane thinning during the pore formation as a function of increased peptide/lipid ratio until the critical ratio at which pores are formed [76]. The depth of the defects in the POPC and Chol/POPC membrane with a mixing ratio of 0.3 caused by the effect of γ-crystallin and measured by AFM in the supported membrane in our studies is ~5 nm, slightly lower than the previously measured POPC and Chol/POPC patches’ membrane thicknesses [62]. The finite water layer sandwiched between the membrane and the mica surface, which helps with lubrication, also contributes to the membrane thickness measured by AFM on SLMs. It has been reported that this water layer thickness is ~1–2 nm [77] and can extend to ~4 nm on very-high-Chol-containing membranes [62]. The spikes and the valley obtained at the bottom of the defect might not correspond to the actual mica surface but the remanent lipid molecules, lipid-γ-crystallin conjugation, or only γ-crystallin aggregates. However, we do not discard the possibility of membrane thinning, too, as the acyl chains of both leaflets exposed to buffer because of pore formation tend to seclude from the water surface, which might result in interdigitation of the acyl chains. The lipid molecules removed due to the association of β_L_- or γ-crystallin on the membrane might be released in the buffer solution; these lipid molecules may produce additional bilayer [78] or micelles [19] to minimize energetics. However, we did not observe any additional bilayer formation in the membrane within our experimental time. The micelles, if formed, might have drifted along with the buffer, and the AFM probe could not detect their existence.

To investigate the interaction of β_L_- and γ-crystallin with SLM, the β_L_- and γ-crystallin solution was incubated with SLM inside the AFM fluid cell. The AFM imaging was performed on the fluid environment. Due to the limitation of the AFM imaging capability to scan only the affixed sample, β_L_- and γ-crystallin present in the solution that are not attached to SLM are not detected and not captured in the AFM image. The lipid molecules removed from the SLM due to the interaction of β_L_- or γ-crystallin with the SLM accompanied by the formation of membrane defects indicates the disintegration or fragmentation of the part of the SLM. The absence of a smooth surface on the bottom of the membrane defects suggests the possibility of lipid–crystallin aggregates on the bottom of the membrane defects. During membrane defects formation, the complex of lipid–crystallin aggregates (complexes of lipid- β_L_ -crystallin or lipid- γ-crystallin) might be released in the buffer solution and float in the vicinity of the SLM or drift along with the buffer; however, the AFM imaging capability can scan only on the affixed sample and such lipid-crystallin complexes if those on the buffer are not detected and not captured on the AFM image. Previously, Garner et al. [79] proposed a cataract development model involving the disintegration and fragmentation of the fiber membrane, and in the cataractous lens, both the membrane and membrane fragments are involved with a γ-crystallin.

Apart from modulating several membrane properties, the association of peptides and proteins with the membrane is also affected by the Chol content in the membrane. The association of beta-amyloid peptide to the membrane is enhanced by an increase in Chol content [80], whereas Chol inhibits the association of blood proteins to the membrane [81]. The association of α-crystallin with membranes made of major eye lens lipids and models of animal and human lens membrane, investigated at varying Chol contents, is found to be inhibited by Chol [36,37]. The presence of Chol and the formation of Chol bilayer domains decreased the hydrophobicity of the membrane and reduced the α-crystallin-membrane association [36,37]. Our AFM experiment in the association of γ-crystallin with a high Chol-containing membrane (Chol/POPC mixing ratio of 1) is completely inhibited within our field of view and experimental time but has a similar effect in a membrane without Chol and a low Chol (Chol/POPC mixing ratio of 0.3). The association of β_L_-crystallin with Chol-containing POPC membrane is distinct from that without Chol. Instead of semi-transmembrane defects formed in Chol free membrane, the association of β_L_-crystallin on the Chol/POPC membrane caused raised rough and granular structures. Additionally, the semi-transmembrane defects formed by β_L_-crystallin observed in the boundary of patches at a Chol/POPC mixing ratio of 0.3 disappeared at a Chol/POPC mixing ratio of 1, suggesting Chol suppresses the formation of semi-transmembrane defects in similar incubation time. Our recent study using the EPR spin-labeling method on β_L_-crystallin association with the model of animal and human eye lens–lipid membranes illustrates that Chol decreases the membrane surface occupied by β_L_-crystallin [46]. It was reported earlier that the modification of membrane composition or the modification of crystallins structure, or the combination of both, might be crucial for altering the affinity of crystallins to associate with the fiber cell plasma membrane of the human lens during aging and cataract development [17]. We also explored the interaction of β_L_-crystallin with the Chol/POPC membrane (Chol/POPC mixing ratios of 0 and 0.3) in different fields of view (i.e., different areas) of the samples and observed similar characteristics and patterns in different regions. We speculate similar characteristics and patterns in different fields of view for the interaction of β_L_-crystallin with Chol/POPC membranes at a mixing ratio of 1 and for the interaction of γ-crystallin with Chol/POPC membranes at mixing ratios of 0 and 0.3; however, further research is needed in this area.

Figure 5 shows the uniform distribution of β_L_- and γ-crystallin on the mica surface. However, β_L_- and γ-crystallin behave distinctly when associated with the membrane. One common observation from the images with defects induced by both β_L_- and γ-crystallin is that the defects are localized rather than uniformly distributed over the membrane. Such localized induced defects might be due to the accumulation of large numbers of β_L_- or γ-crystallin subunits during or around membrane association time. An initial association of a single crystallin subunit to the membrane might trigger the association of surrounding crystallin subunits. The initial association of the crystallin subunit might expose the hydrophobic core of the membrane, which triggers the association of other crystallin subunits and cascades the association. A similar cascade association of α-crystallin on POPC membrane was seen previously [35]. It has been reported earlier that β-crystallin subunits interact with each other via hydrophobic interactions [82] and that domains in γ-crystallin interact via hydrophobic interactions [83]. During these hydrophobic interactions between the β_L_-crystallin subunits or between domains in γ-crystallin, one of the subunits or domains might associate with the membrane via hydrophobic interaction, which might act as a seed for the accumulation and localized association of crystallins in the membrane, resulting in membrane defects. Several studies have shown surface-induced protein denaturation [84,85,86]. It has been reported that the interaction of the proteins with the interfaces represents a significant factor in protein aggregate formation [86]. Additionally, studies show that the protein’s hydrophobic core is disrupted, and the protein is destabilized due to protein interaction with the surface [84]. When the β_L_- and γ-crystallin interact with the membrane, the opening of the hydrophobic domain interface in γ-crystallin and the hydrophobic subunit interface in β_L_-crystallin is possible, leading to more substantial misfolding of the β_L_- and γ-crystallin, which might result in association with the membranes, promoting increased accumulation of β_L_- and γ-crystallin in the membrane, resulting in membrane defects. This aggregation of crystallins in the membrane, followed by membrane defect formation, is likely to obstruct the path of light and scatter light significantly more than the association of single crystallin subunits or domains in the membrane. Interestingly, β_L_-crystallin forms semi-transmembrane defects, and γ-crystallin forms transmembrane defects. We speculate that during interactions of γ-crystallin with the membranes, more hydrophobic residues are exposed on the surface, resulting in strong interaction of γ-crystallin with the phospholipid membrane, forming transmembrane defects. In contrast, in the case of β_L_-crystallin interaction with the membrane, the hydrophobic residue exposed on the surface might be lesser than γ-crystallin, resulting in relatively weaker interaction with the phospholipid membrane compared to γ-crystallin, resulting in semi-transmembrane defects; however, further research is needed in this area. With the addition of Chol on the phospholipid membrane, the interaction of β_L_-crystallin is different and diminishes the semi-transmembrane defects. On the other hand, high Chol completely suppresses the interaction between γ-crystallin and phospholipid membrane within our range of observation, resulting in a defect-free membrane. Previously, Raguz et al. demonstrated that the surface hydrophobicity of the POPC membrane decreases with increased Chol content in the membrane [87]. Based on these observations, our experimental result shows that the interaction between β_L_- and γ-crystallins with the membrane might be directed by hydrophobic interaction. Several factors, including lipid (PLs and sphingolipids) and Chol composition, β- and γ-crystallin modifications, membrane lipid and Chol oxidation, and intrinsic membrane proteins might guide the actual interaction of β-and γ-crystallins in the lens plasma membrane in vivo. To achieve a better understanding of the roles of Chol and lipid composition in β-and γ-crystallin association with lens membrane, the study on the role of individual eye lens lipid and the lipid and Chol composition resembling animal and human lens membranes—a lipid extract from different regions of the lens, recombinant β-and γ-crystallin, mutated β-and γ-crystallin—might provide deeper insight into β-and γ-crystallin’s association with the membrane and membrane defect formation, which we plan to investigate in the future.

Lens membrane consists of three major components, i.e., lipids (PLs and sphingolipids), Chol, and intrinsic proteins [32,88,89]. Previously, Su et al. [90] investigated the interaction of αA-crystallin with bovine and human lens membranes containing intrinsic proteins and suggested that αA-crystallin might interact exclusively with phospholipids of the lens membrane. Similarly, the studies on the lens membranes isolated from different regions of the human lens showed that β- and γ-crystallin are tightly bound with lens membranes with increasing age, suggesting that such binding alters membrane properties [21]; however, whether the primary binding sites of β- and γ-crystallin in lens membranes are lipids or intrinsic proteins is unclear. In the present study, we have investigated the interaction of β_L_- and γ-crystallin with artificial Chol phospholipid membranes and found that Chol suppresses the formation of membrane defects. Future studies on the interaction of β_L_- and γ-crystallin with the lens membrane containing intrinsic proteins would further elucidate the interaction of β_L_- and γ-crystallin with the lens membrane.

The contribution by lipids and Chol of lens membrane to the scattering of light was thoroughly discussed [91,92,93,94]. However, the scattering of light by the human lens is a very complicated phenomenon with difficulty in separating the contributions to light scattering from different lens components because of the mutual interaction between different components [21,32,44,46,91,95]. The light scattering in the case of the membrane originates from the bulk of the membrane and the structural distortion at the membrane surface [67,96], where the contributions to the surface scattering come from the irregularities of the membrane surface [96]. We hypothesize that the membrane defects observed in Figure 1, Figure 2, Figure 3, Figure 4 and Figure 5 contribute to the light scattering. Eye lenses consist of 1000 to 3000 layers of fiber cells [97]. Light travels across thousands of cell membranes, and the scattering of light at each defect’s membrane surface is amplified. Thus, we hypothesize that Chol suppresses the formation of membrane defects, reducing the scattering of light by the lens and helping maintain lens transparency. Research is needed to test this hypothesis.

For the age-matched clear and cataractous lenses of 61–70-year-old donors, the Chol/lipid ratios for cortical and nuclear membranes of the clear lens are 1.8 and 4.4, respectively [60]. In contrast, the Chol/lipid ratio of cortical and nuclear membranes of cataractous lens are 1.14 and 1.45, respectively [60]. Similarly, for the age group of 73–80-year-old donors, the Chol/lipid ratio for clear and cataractous lens membranes is 3.1 and 1.7, respectively [64]. Our results show that at a Chol/POPC mixing ratio of 1, in the case of γ-crystallin, Chol completely suppresses the formation of transmembrane defects within our experimental view and experimental time; however, in the case of β_L_-crystallin, Chol suppresses the formation of semi-transmembrane defects, leaving the raised structure in the boundary and granular form in the center within our experimental view and experimental time. There are four major lipids (i.e., PC, SM, PE, and PS) that build the lipid bilayer portion of the eye lens membrane [56], and our recent EPR spin-labeling studies on the interaction of β_L_-crystallin with the model of porcine lens–lipid (MPLL), the model of mouse lens–lipid (MMLL), and the model of human lens–lipid (MHLL) at Chol/lipid ratios of 0 and 0.3 show that binding of β_L_-crystallin is larger in PC dominant MPLL and MMLL membranes compared to SM dominant MHLL membranes [46]. Additionally, Chol inhibits the binding of β_L_-crystallin to Chol/MPLL, Chol/MMLL, and Chol/MHLL membranes differently [46]. With aging and cataract formation, β- and γ-crystallin undergo post-translational modifications [63], which might increase the binding of β- and γ-crystallin with the lens membrane; however, further research is needed in this area. Based on these observations and findings in this manuscript, we speculate that the need for high Chol content (i.e., Chol/lipid ratio as high as 4.4) in the human lens membrane might be necessary to prevent the binding of β_L_- and γ-crystallin to the lens membrane, suppressing the membrane defect formation, and promoting lens membrane and lens cytoplasm homeostasis, likely protecting against light-scattering cataract formation.

## 4. Materials and Methods

### 4.1. Materials

Bovine eye lenses were purchased from Pel-Freez Biologicals (Rogers, AZ, USA) and were stored at −80 °C immediately after receiving them. POPC lipid dissolved in chloroform, and Chol in powdered form was purchased from Avanti Polar Lipids (Birmingham, AL, USA) and used without further purification. NaN_3_, MgCl_2_, HEPES, and NaCl of analytical grades were purchased from Sigma Aldrich (St. Louis, MO, USA). Tris base was purchased from Fisher Bioreagents. The elution buffer contained 20 mM Tris-HCl, 150 mM NaCl, and 1 mM NaN_3_ with pH 7.9. Buffer A contained 10 mM HEPES, 150 mM NaCl with pH 7.4 with 10 mM MgCl_2_, while buffer B contained 10 mM HEPES, 150 mM NaCl, pH 7.4. All small unilamellar vesicle (SUV) suspension solutions were prepared in buffer A.

### 4.2. β_L_-and γ-Crystallin Isolation from Bovine Lens

A single bovine lens was separated into the cortical and nuclear regions using a scalpel depending on the tissue consistency after decapsulation [35]. Soluble proteins were extracted from the cortex using the previously described protocol [98]. Briefly, the homogenized cortical tissues in the elution buffer were pelleted by centrifuging at 18,000 RCF for 15 min at 4 °C (Beckman Coulter, Brea, CA, USA), and only supernatant was taken for protein isolation. A total of 5 mL filtered (0.22 µm pore size) supernatant was loaded onto a Hiload 16/600 Superose 6 pg gel filtration column connected in AKTA go protein purification system, eluted at a flow rate of 1 mL/min, and protein fractions were monitored at 280 nm absorbance. All the protein fractions were collected in a rotatory fraction collector. The fourth and fifth peaks, corresponding to β_L_-crystallin (low molecular weight β-crystallin) and γ-crystallin, respectively [98], were used for our experiments. As described previously [99,100], β_L_- and γ-crystallin fractions were further purified using the Sephacryl S-200 HR and Sephacryl S-100 HR column at room temperature, respectively. After re-chromatography, both protein solutions were further concentrated using Amicon Ultra-15 filters by centrifuging at 5000 RPM at 4 °C and stored at −80 °C until further use. SDS-PAGE was used to verify the purity of β_L_- and γ-crystallin. The concentrations of the β_L_- and γ-crystallin were estimated using a nanodrop one^C^ spectrophotometer (Thermo Fisher Scientific, Madison, WI, USA), taking account of molecular weight and extinction coefficient of corresponding crystallin proteins. The average molecular weight and extinction coefficient of β_L_-crystallin estimated using the ProtParam tool on the Expasy server [101] was 24.31 kDa and 55409 M^−1^cm^−1^, respectively, where the average was taken from seven β-crystallin subunits (βB1, βB2, βB3, βA1, βA2, βA3, βA4). Previously, it was reported that β_L_ crystallin extracted from the cortex of a bovine lens consists of these seven β crystallin subunits [8]. Similarly, the average molecular weight and extinction coefficient of γ-crystallin was estimated using the ProtParam tool on the Expasy server [101] was 21.04 kDa and 44730 M^−1^cm^−1^, respectively, where the average from eight γ-crystallins (γA-, γB-, γC-, γD-, γE-, γF-, γN-, and γS) was taken. These different γ-crystallins were reported earlier in bovine lens [65,66].

### 4.3. Supported Lipid Membrane (SLM) Preparation and Interaction with β_L_-and γ-Crystallin

As described previously, the SUVs were prepared via probe tip sonication of multilamellar vesicles prepared using the rapid solvent exchange (RSE) method [61,62]. SLM made of POPC and POPC plus Chol with different Chol content was prepared as described previously [62]. Briefly, around 500 µL of 0.1–0.2 mg/mL SUVs suspension solution in buffer A was deposited on a freshly cleaved mica disk under the AFM head consisting of fluid cell accessory. After 20–35 min of incubation, rinse buffer (buffer B) was dispensed through one of the inlets in the fluid cell to replace the divalent-salt-containing buffer so that the effect of divalent salt, if any, would be minimal. After verifying the uniformity of SLM and acquiring a control image, in each experiment, 400 µL of 0.025 mg/mL of β_L_- or γ-crystallin was passed via the inlet of the fluid cell to incubate with SLM, of which ~75 µL of β_L_- or γ-crystallin solution remained in the well created by a silicon O-ring in the fluid cell. The radius of the silicon O-ring in the fluid cell was ~0.5 cm; thus, ~75 µL of 0.025 mg/mL of β_L_- or γ-crystallin was incubated with the SLM with a surface area of ~0.785 cm^2^ exposed for the interaction of β_L_- or γ-crystallin with the SLM. To observe the distribution and structure of the studied crystallin proteins in the absence of the membrane, approximately 10 µL of 0.025 mg/mL of β_L_- or γ-crystallin solution was deposited in a freshly cleaved mica disk and dried openly at ambient temperature, five times subsequently washed with ~500 µL water, re-dried, and scanned using AFM. All the experiments were performed at a room temperature of ~21 °C and a room humidity of ~45%. It has been reported that ambient factors can influence AFM measurements [102]. The temperature of the fluid cell might be slightly higher than the room temperature due to the heated electronics and the laser in the AFM setup; however, all our experiments are performed on similar conditions, ensuring the reproducibility of the results. All of our AFM measurements for the interaction of SLM with β_L_- or γ-crystallin were performed in a fluid phase in which the silicon O-ring in the fluid cell seals the fluid containing SLM and β_L_- or γ-crystallin avoiding contact with the air. For the air AFM imaging of dried β_L_- or γ-crystallin on the mica surface, we expect the humidity in the fluid cell to be similar or slightly different than the room humidity. Each experiment was repeated at least three times to ensure the reproducibility of the results.

### 4.4. AFM Measurement and Hydrodynamic Radius Estimation

A Bruker multimode VIII AFM equipped with a Nanoscope V controller and J-Scanner was used for all image acquisitions and to capture force curves. A commercially available AFM probe (DNP-S) with a nominal tip radius of 10 nm and nominal spring constant of 0.35 N/m were attached to the fluid cell or standard air probe holder for imaging. Peak force–quantitative nanomechanical mapping (PF-QNM) mode in fluid or air was used to capture images at a 1 Hz scan rate and 384 × 384 samples per line. For fluid imaging, the peak force setpoint, amplitude, and frequency were set at 600 pN, 25 nm, and 2 kHz, respectively, while for imaging in air, peak force setpoint, amplitude, and frequency were set at 2 nN, 125 nm, and 2 kHz, respectively. The inbuilt thermal noise tool was used to calibrate spring constant before each experiment and was obtained in the range of 0.4 N/m to 0.6 N/m. Similarly, the tip end radius was estimated to be ~13 nm by analyzing height images of the Ti- roughness characterizer (Model: PFQNM-SMPKIT, Bruker, Germany). The inbuild point and shoot function was used to capture force curves at least 200 points with minimal separation of 100 nm between the points in the captured images. Images were flattened via first- or second-order fit using Nanoscope analysis 1.9 (Bruker, Santa Barbara, CA, USA) software and further processed using a homebuilt script using Matlab (Mathworks, MA, USA). It has been reported that AFM probes stored in gel boxes result in the contamination of the probes by a thin layer of silicone oil [103,104], and the cleaning procedure for AFM probes has been reported [104,105]. We have used a similar cleaning procedure described in the previous studies [105]. Before each experiment, the AFM fluid cell was thoroughly rinsed with phosphate-free detergent (Catalog number 27959, Thermo Scientific) and ultrapure water, dried with a stream of nitrogen gas, affixed with the AFM probe, and rinsed three to four times with ultrapure water and ethanol alternatively. The fluid cell with the probe inserted and without silicon O-ring was then dried with a stream of nitrogen gas. After performing five to six experiments, the cleaning procedure also involved rinsing the AFM fluid cell with chloroform while having a probe inserted and without a silicon O-ring.

We used DLS to estimate the R_h_ and % Pd of the β_L_- and γ-crystallin where % Pd gives the measure of the homogeneity or heterogeneity of the particles in the solution. The concentrations of β_L_- and γ-crystallin used for DLS measurements are 0.25 mg/mL. The DynaPro NanoStar instrument (Wyatt Technology Corp., Santa Barbara, CA, USA) and regularization methods (Dynamics software, version 7) were used to estimate the R_h_.

### 4.5. Statistics

The measurements from at least three independent experiments were used to estimate the mean and standard deviation of the depth and area of the membrane defects. The depth of the defects was estimated using the rotating box function of Nanoscope Analysis 1.9 software in at least four boundary locations of the membrane defects. The area of the membrane defects was estimated using the inbuilt regionprops function in Matlab. The shape of the membrane defects was accessed from at least three independent experiments and reported.

## 5. Conclusions

We used the fluid AFM approach to study the association of eye lens two major crystallin proteins—i.e., β_L_- and γ-crystallin—with POPC membrane containing various Chol concentrations. Our AFM experiments provide the first evidence of visual detection of the β_L_- and γ-crystallin association with the membrane. We demonstrated that β_L_- and γ-crystallin are prone to membrane association and exhibit distinct membrane modulation effects inducing semi-transmembrane and transmembrane defects, respectively. However, high Chol in the membrane prevents the association of β_L_- and γ-crystallin to the membrane and suppresses the formation of membrane defects, likely maintaining lens membrane homeostasis and protecting against cataract formation. Crystallins (α-, β-, and γ-crystallin) account for about 90% of the soluble lens proteins in the cytoplasm along with other lens proteins [106,107]. With aging, the level of soluble proteins declines in the cytoplasm with the corresponding increase in membrane-bound proteins leading to light scattering and cataract formation [17,18]. Several β- and γ-crystallin mutations are associated with different cataract types [63], and it may be possible that mutated β- and γ-crystallin might increase membrane associations and membrane defect formation. Additionally, it has been reported that the Chol content in cataractous lens membranes is significantly lower than the age-matched transparent lenses [60,64], suggesting that low Chol in cataractous lens membranes might be responsible for the association of β- and γ-crystallin in the membranes and the formation of membrane defects. Further studies on the interactions of β- and γ-crystallin with the membranes focusing on the cataract associated β- and γ-crystallin mutations and the membranes prepared with lipid extracts from clear and cataractous lenses would elucidate the mechanism of light scattering and cataract formation.

## Data Availability

Not applicable.
